# Environmental Behaviors, Ecological Risks, and Toxic Mechanisms of Emerging and Legacy Contaminants in China: From Distribution to Management

**DOI:** 10.3390/toxics14030263

**Published:** 2026-03-17

**Authors:** Weiying Feng, Chenglian Feng, Bingli Lei

**Affiliations:** 1 School of Materials Science and Engineering, Beihang University, Beijing 100191, China; fengweiying@buaa.edu.cn; 2 State Key Laboratory of Environmental Criteria and Risk Assessment, Chinese Research Academy of Environmental Sciences, Beijing 100012, China; 3 Institute of Environmental Pollution and Health, College of Environmental and Chemical Engineering, Shanghai University, Shanghai 200444, China; leibingli@126.com

## 1. Introduction

In recent years, the accelerated pace of industrialization and urbanization has exacerbated the coexistence and combined pollution of both emerging and legacy contaminants in aquatic environments, posing potential threats to ecosystem integrity and human health. This Special Issue, entitled “Occurrence and Environmental Risks of Organic Pollutants in Aquatic Environment”, compiles eight research articles focusing on the environmental behaviors, toxic effects, and risk assessment of various contaminants, including polycyclic aromatic hydrocarbons (PAHs) (contribution 1), per- and polyfluoroalkyl substances (PFASs) (contribution 2), organophosphate flame retardants (OPFRs) (contribution 2), phosphorus fractions in lake sediments (contribution 3), microplastics (contribution 4), disinfection byproducts (DBPs) (contribution 5), the plasticizer DEHP (contribution 6), pesticides (contribution 7), and the persistent organic pollutant dieldrin (contribution 8).

Through the application of field monitoring, bioassays, bioinformatics, metabolomics, and species sensitivity distribution models, these studies systematically elucidate the multimedia distribution, bioaccumulation potential, and toxicological effects of these pollutants, including neurotoxicity, immunotoxicity, oxidative stress, and ecological risks in aquatic organisms. The findings provide a robust scientific basis for the integrated management of both emerging and traditional pollutants in aquatic environments. Moreover, they directly contribute to the refinement of global water quality criteria and the prioritization and precise identification of high-risk contaminants while offering key technical guidance for lake eutrophication control, drinking water source protection, and watershed-scale ecological risk management.

## 2. Overview of Published Articles

To begin with, Li et al. systematically investigated the distribution and ecological risks of polycyclic aromatic hydrocarbons (PAHs) and pesticides in the surface water and sediments of the Danjiangkou Reservoir. The results showed that the total concentration of PAHs (ΣPAHs) in the water column ranged from 64.64 to 868.23 ng/L, with low-molecular-weight PAHs (e.g., naphthalene, fluorene, and phenanthrene) being the predominant compounds. Pesticides were mainly composed of organophosphorus compounds, with ΣOPPs concentrations ranging from 2.62 to 71.14 ng/L. In sediments, ΣPAHs concentrations ranged from 0.01 to 2.93 ng/g, dominated by high-molecular-weight PAHs, while organophosphorus pesticides such as malathion and chlorpyrifos exhibited relatively higher concentrations. Ecological risk assessment indicated that phenanthrene, malathion, and other contaminants posed moderate to high risks to aquatic organisms, particularly to daphnids, whereas human health risks were relatively low. The study concludes that although the Danjiangkou Reservoir is generally safe as a drinking water source, certain organic pollutants present potential threats to the aquatic ecosystem, underscoring the need for continuous monitoring (contribution 1).

Building on this, Sun et al. further characterized the occurrence of 18 per- and polyfluoroalkyl substances (PFASs) and 9 organophosphate flame retardants (OPFRs) in the upper reaches of the Yangtze River. The results revealed that total PFAS concentrations ranged from 16.07 to 1231.11 ng/L, with ultra-short-chain PFASs (e.g., TFMS) being the most abundant. Total OPFR concentrations ranged from 17.36 to 190.42 ng/L, with halogenated OPFRs such as TCPP dominating. Pollutant levels were higher in the main stream than in tributaries, and contamination loads were greater during the rainy season. Ecological risk assessment indicated low risk at most sites, though moderate risks from OPFRs were identified in certain areas (e.g., Leshan and Chongqing), with wastewater discharge being a key source. Health risk assessment showed acceptable non-carcinogenic risks, but potential carcinogenic risks from OPFRs were identified for adults in the Leshan section. The study recommends strengthening regulatory oversight of emerging contaminants and improving wastewater treatment technologies (contribution 2).

In addition to organic pollutants, the transport and transformation of nutrients remain a central focus in aquatic environmental research. Wu et al. investigated the relationship between phosphorus fractions in sediments and dissolved organic matter (DOM), human activities, and ecological characteristics in three plateau lakes of Yunnan Province: Dianchi Lake (eutrophic, algae-dominated), Erhai Lake (mesotrophic, grass–algae mixed), and Yangzonghai Lake (mesotrophic, algae-dominated). The results demonstrated that human activities significantly promoted the accumulation of immobile phosphorus fractions (e.g., NaOH-rP and HCl-P) in sediments, while showing no significant effect on mobile phosphorus. Mobile phosphorus content was highest in Dianchi Lake, exceeding that in Erhai and Yangzonghai Lakes by 40.6% and 278.3%, respectively, indicating that the shift from macrophyte-dominated to algae-dominated states enhances phosphorus mobility. DOM influenced phosphorus mobility primarily by promoting the adsorption of iron-bound phosphorus (BD-P) and organic phosphorus (NaOH-nrP). The study emphasizes the need for differentiated management strategies tailored to lakes of varying trophic states to control internal phosphorus release (contribution 3).

Building on the understanding of pollutant distribution and transport, elucidating their degradation processes is essential for assessing environmental fate. Cao et al. employed bibliometric analysis to systematically review research progress on the biodegradation of microplastics from 2012 to 2022. The findings indicate that research hotspots have centered on functional microorganisms—such as bacteria from the phyla Proteobacteria and Firmicutes, and fungi from Ascomycota—alongside the mechanisms of action of their secreted degradative enzymes, including hydrolases and oxidases. Microorganisms degrade microplastics through colonization, biofilm formation, and enzymatic breakdown. Future research should expand the focus to include degradation mechanisms in soil and atmospheric environments and further explore the molecular degradation pathways of these functional microorganisms (contribution 4).

In addition to degradation processes, the direct toxic effects of pollutants and their underlying molecular mechanisms warrant in-depth investigation. Zhang et al. elucidated the toxic mechanisms of the disinfection byproduct 2,6-dichloro-1,4-benzoquinone (2,6-DCBQ) on *Microcystis aeruginosa*. The study revealed that 2,6-DCBQ inhibits algal growth, reduces photosynthetic pigment and protein contents, induces oxidative stress (elevated reactive oxygen species levels and altered antioxidant enzyme activities), and disrupts cell membrane integrity. Concurrently, it stimulated the synthesis and release of microcystin-LR (MC-LR) and regulated the expression of related genes (mcyA, mcyD, mcyH). Non-targeted metabolomics identified 208 differentially abundant metabolites, which were primarily enriched in pathways associated with ABC transporters, two-component systems, and folate biosynthesis. The study concludes that 2,6-DCBQ exerts comprehensive toxicity on *M. aeruginosa* through interference with energy metabolism, induction of oxidative damage, and promotion of toxin release, providing a theoretical basis for assessing the aquatic ecological risks of such emerging disinfection byproducts (contribution 5).

Similarly, the toxic mechanisms of plasticizers have garnered considerable attention. Meng et al. investigated the neurotoxicity and potential carcinogenic mechanisms of the plasticizer di-2-ethylhexyl phthalate (DEHP) using bioinformatics analyses and zebrafish toxicology assays. The results demonstrated that the circadian regulator PER3 was significantly downregulated in glioblastoma multiforme (GBM) tissues and was closely associated with immune cell infiltration, immune checkpoint genes, and oncogenes. Low PER3 expression was significantly correlated with poor prognosis and advanced clinical stage, exhibiting high diagnostic value (AUC = 0.916). Further zebrafish experiments revealed that DEHP exposure led to developmental abnormalities, increased anxiety-like behaviors, and significant downregulation of PER3 gene expression in brain tissue. The study suggests that PER3 is a potential novel diagnostic marker for GBM, and that DEHP may exert carcinogenic effects by disrupting circadian gene expression (contribution 6).

In real-world environments, pollutants often co-exist as complex mixtures, rendering their combined toxic effects considerably more intricate. Liu et al. investigated the acute toxicity, neurotoxicity, immunotoxicity, and behavioral effects of deltamethrin (DM) and sulfamethoxazole (SMX) on adult zebrafish. The results showed that the 96 h LC50 of DM was 4.84 μg/L, indicating high acute toxicity; however, following combined exposure with SMX, the LC50 increased to 11.32 μg/L, suggesting that SMX may partially mitigate the toxicity of DM. Exposure to DM significantly reduced the levels of GABA, 5-HT, and AChE in the brain, induced oxidative stress (elevated SOD and GST activities, decreased CAT and GPX activities), and suppressed the expression of immunoglobulin genes (IgM, IgD, IgZ), while upregulating the pro-inflammatory cytokine IL-8. Combined exposure partially restored AChE activity and feeding behavior. This study reveals that DM exhibits potent neurotoxicity and immunotoxicity, and that the co-presence of SMX can modulate these toxic effects, highlighting the need to consider chemical interactions in environmental risk assessments (contribution 7).

To translate toxic effects into actionable management thresholds, the derivation of scientifically sound water quality criteria is essential. Xie et al. employed a tissue residue approach, combined with a bioaccumulation factor (BAF) model, to derive water quality criteria for dieldrin aimed at protecting aquatic organisms and wildlife. By screening tissue-based toxicity data for aquatic organisms, birds, and mammals, tissue reference values were obtained using the species sensitivity distribution (SSD) method. These values were then back-calculated to corresponding water concentrations using an estimated BAF of 58,884.37 L/kg. The results indicated that the criteria for protecting aquatic life and aquatic-dependent wildlife are 3.86 ng/L and 1.4 ng/L, respectively. Risk assessment revealed that dieldrin residues in some surface waters of China pose potential ecological risks to aquatic organisms and birds. The study recommends adopting the tissue residue approach to derive water quality criteria for bioaccumulative pollutants, thereby ensuring more effective protection of aquatic ecosystems (contribution 8).

In summary, these studies collectively reveal the complex reality of aquatic environments, characterized by a diverse array of pollutants, multiple sources, and varied toxicological mechanisms. For instance, research on the Danjiangkou Reservoir demonstrated that, despite relatively low overall concentrations of PAHs and pesticides, certain organophosphorus pesticides pose moderate to high risks to aquatic organisms (contribution 1). Investigations in the upper reaches of the Yangtze River revealed the widespread occurrence of PFASs and OPFRs, with wastewater discharge serving as a significant source and potential carcinogenic risks identified at specific sites (contribution 2). Studies on plateau lakes such as Dianchi and Erhai further indicated that human activities substantially influence phosphorus speciation and mobility in sediments, and that the ecological shift from macrophyte-dominated to algae-dominated states exacerbates the risk of endogenous phosphorus release (contribution 3). Furthermore, research on the combined toxicity of deltamethrin and sulfamethoxazole highlighted that interactions between co-occurring pollutants can alter the toxic effects of individual compounds, underscoring the necessity of considering mixture effects in risk assessments (contribution 7). More importantly, the derivation of water quality criteria for dieldrin using a tissue residue approach (contribution 8) addresses the limitations of traditional water-based criteria in adequately protecting against bioaccumulative pollutants, thereby offering a novel strategy for the precise control of high-risk persistent contaminants.

## 3. Future Perspectives and Concluding Remarks

Collectively, the research presented in this Special Issue has not only deepened the understanding of the fate and toxic mechanisms of both legacy and emerging contaminants in aquatic environments but has also provided a critical scientific basis for refining China’s water quality criteria system, optimizing pollutant monitoring and risk assessment methodologies, and formulating differentiated watershed-scale pollution control strategies. These studies have collectively advanced a paradigm shift from single-pollutant assessment to combined pollution evaluation, from external exposure concentrations to internal exposure doses, and from water-based criteria to tissue-residue-based criteria, thereby laying a solid foundation for the precise protection of aquatic ecosystems and the achievement of sustainable development goals.

Future research should be directed toward the following key areas: first, further investigation into the combined toxic mechanisms of multiple pollutants under co-exposure scenarios, with a focus on synergistic or antagonistic interactions and their integrated effects on the nervous, immune, and metabolic systems of aquatic organisms. Second, deeper exploration of molecular mechanisms underlying toxicity, integrating multi-omics technologies to elucidate toxicological pathways and identify robust biomarkers. Third, the refinement of water quality criteria derivation methods based on tissue residue and bioaccumulation assessments to enhance the precise control of persistent and bioaccumulative pollutants. Fourth, the expansion of research matrices from the aqueous phase to sediments, soils, and the atmosphere, thereby build a multimedia integrated ecological risk assessment framework. Ultimately, these efforts will establish full-chain scientific support—from pollutant identification to toxic mechanism elucidation and management application—providing both theoretical foundations and technical guidance for aquatic ecosystem health and sustainable watershed management.

## Data Availability

Data sharing is not applicable.

